# DNA Dosimetry Assessment for Sunscreen Genotoxic Photoprotection

**DOI:** 10.1371/journal.pone.0040344

**Published:** 2012-06-29

**Authors:** André Passaglia Schuch, Juliana Carvalhães Lago, Teiti Yagura, Carlos Frederico Martins Menck

**Affiliations:** 1 Department of Microbiology, Institute of Biomedical Sciences, University of São Paulo, São Paulo, Brazil; 2 Natura Inovação e Tecnologia de Produtos LTDA, São Paulo, Brazil; Maastricht University Medical Center, The Netherlands

## Abstract

**Background:**

Due to the increase of solar ultraviolet radiation (UV) incidence over the last few decades, the use of sunscreen has been widely adopted for skin protection. However, considering the high efficiency of sunlight-induced DNA lesions, it is critical to improve upon the current approaches that are used to evaluate protection factors. An alternative approach to evaluate the photoprotection provided by sunscreens against daily UV radiation-induced DNA damage is provided by the systematic use of a DNA dosimeter.

**Methodology/Principal Findings:**

The Sun Protection Factor for DNA (DNA-SPF) is calculated by using specific DNA repair enzymes, and it is defined as the capacity for inhibiting the generation of cyclobutane pyrimidine dimers (CPD) and oxidised DNA bases compared with unprotected control samples. Five different commercial brands of sunscreen were initially evaluated, and further studies extended the analysis to include 17 other products representing various formulations and Sun Protection Factors (SPF). Overall, all of the commercial brands of SPF 30 sunscreens provided sufficient protection against simulated sunlight genotoxicity. In addition, this DNA biosensor was useful for rapidly screening the biological protection properties of the various sunscreen formulations.

**Conclusions/Significance:**

The application of the DNA dosimeter is demonstrated as an alternative, complementary, and reliable method for the quantification of sunscreen photoprotection at the level of DNA damage.

## Introduction

The range of sunlight corresponding to UVB (290–320 nm) and UVA (320–400 nm) wavelengths is the cause of numerous adverse skin effects, such as sunburn, skin aging and the induction of skin cancer [1]. The observation that squamous and basal-cell carcinomas develop almost exclusively in sunlight-exposed areas in individuals who rarely tan or burn easily is clear evidence of the relationship between sun exposure and the occurrence of skin cancers [2]–[3]. These adverse effects are even more evident when considering the induction of the often fatal cutaneous malignant melanoma. This disease has recently displayed a significant increase with an incidence that has more than doubled over the past 25 years [4].

Sunlight carcinogenesis is linked to a chain of events following exposure that includes the induction of DNA damage and subsequent mutation [5]. Notably, different wavelengths of UV light induce several types of DNA damage [6]. Direct excitation of DNA molecules, mainly by UVB wavelength light, gives rise to well-known photochemical reactions that lead to the formation of DNA photoproducts that are known as cyclobutane pyrimidine dimers (CPDs) and pyrimidine (6–4) pyrimidone photoproducts (6-4PPs) [7]–[9].

Apart from the direct induction of DNA photoproducts, UV radiation can also indirectly give rise to DNA damage through UVA photon absorption by other chromophores and the subsequent generation of reactive oxygen species [2]. Oxidised DNA bases, such as 7,8-dihydro-8-oxoguanine, have often been proposed to be pre-mutagenic lesions in UVA mutagenesis [2], [10]–[14]. In contrast, recent studies suggest that CPD is the most relevant UVA-induced type of DNA lesion observed in various biological models, such as purified DNA samples [9], cultured cells [15] and whole-skin explants [6]. Recently, *in vivo* assaying revealed that UVA1 (340–400 nm) induces CPDs in the skin of healthy volunteers, indicating that UVA radiation may be more carcinogenic than previously assumed [16].

With regards to public health-care, photoprotection has become a topic for increased attention, and the use of sunscreen lotions is widely considered to be one of the main defense mechanisms against the harmful effects of UV radiation. The evaluation of protection efficiency occurs mainly through the induction of erythema in human skin and is expressed as a sun protection factor (SPF). In addition to SPF, other UVA protection parameters, such as *in vivo* Persistent Pigment Darkening (PPD) and *in vitro* UVA-PF, have recently been introduced. An important issue that relates to biological relevance is that these parameters do not reflect other deleterious effects of UV radiation, such as immunosuppression, photoaging and carcinogenesis [1]. Furthermore, it is evident that personal exposure time is directly influenced by the labelled SPF, with people using sunscreens to prolong intentional sun exposure [17].

The high prevalence of UV-related diseases makes the need for methodologies that use molecular approaches critical to complement the current approaches that evaluate sun protection and to accurately inform consumers as to the photoprotection efficacy of commercial sunscreens. Because DNA is the main target of solar UV-radiation in living cells and DNA photoinduced lesions are a prerequisite for triggering several biological skin effects, evaluation of the protection that is provided against DNA-induced damage would be a worthy parameter to complement the limitations of current approaches. Ample evidence of DNA protection offered by sunscreen exists and has been demonstrated using various models, such as the skin of human volunteers [18], mice [19], human skin explants [1], a reconstructed human skin model [18], [20], and *in vitro* cultured human cells [21]–[23]. In this study, a simple and robust *in vitro* approach is proposed for rapid screening of photoprotection against the induction of several types of DNA lesions after exposure to simulated solar UV radiation. This strategy is based on the exposure of a highly UV-transparent DNA-based biosensor to a solar simulator [9]. The Sun Protection Factor for DNA (DNA-SPF), calculated by using the DNA repair enzymes *E. coli* formamidopyrimidine-DNA glycosylase (Fpg; recognizes mainly oxidatively generated damage in purines) and T4 bacteriophage endonuclease V (T4-endo V; specific for CPDs), includes the fold protection provided by the sunscreen against the induction of both CPDs and oxidised DNA bases compared with unprotected DNA samples.

By using a DNA dosimeter that is complementary to the labelled SPF, it was possible to demonstrate the important DNA damage protection profiles that are provided by various commercial products and sunscreen formulations following exposure to daily simulated sunlight, which is comparable to prevailing tropical conditions.

## Results

### DNA Photoprotection Properties of five Different Brands of Commercially Available SPF 30 Sunscreens

Exposure of the DNA-based biosensor was carried out in a solar simulator to mimic two hours of sun exposure on a clear-sky summer day in São Paulo (23°32′S; 46°38′W), the largest city in Brazil, and one of the most populous cities in the world. To do so, DNA dosimeters were irradiated for 2∶06 hours, which correspond to 300,000 J/m^2^ of simulated solar UV radiation. This simulated irradiation was therefore consistent with the environmentally observed UV dose under similar conditions in which the natural solar UV dose measured from 11∶00 a.m. to 13∶15 p.m. (2∶15 hours) corresponds to 300,861 J/m^2^.

**Figure 1:**
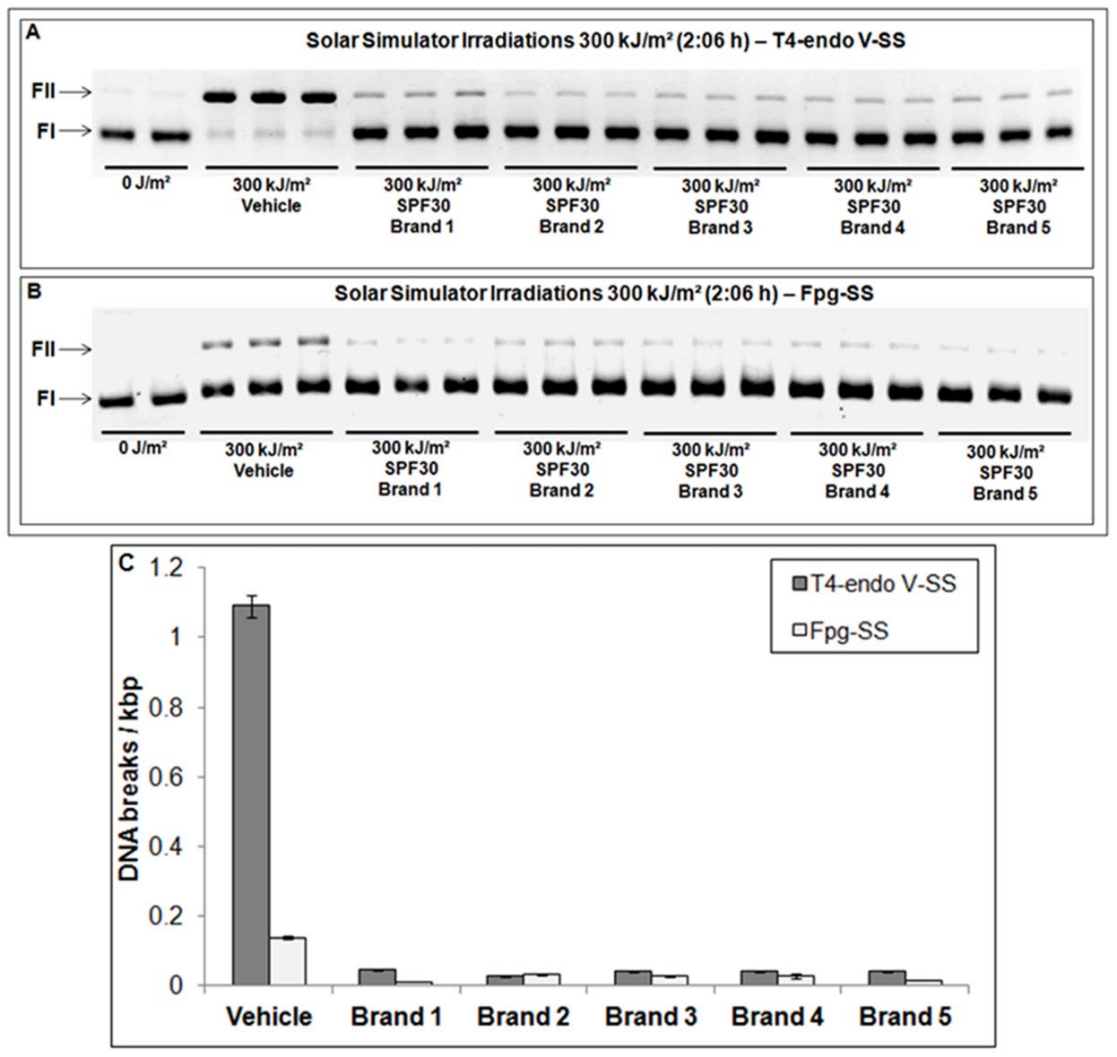
Determination and quantification of DNA lesions after exposure to a solar simulator. Treatment with the DNA repair enzymes T4-endo V (**A**) or Fpg (**B**). The number of DNA lesions induced by simulated sunlight in the absence (**vehicle**) or presence of five different commercially available SPF 30 sunscreens (**C**). FI (supercoiled plasmid DNA bands); FII (open-circular relaxed DNA bands); T4-endo V-SS (T4-endo V sensitive sites – CPDs); Fpg-SS (Fpg sensitive sites – oxidised DNA bases). The average and standard deviation from three independent experiments are shown. The experiments were performed in triplicate.

Initially, five brands of commercial SPF 30 sunscreens were homogeneously applied to the surface of the DNA dosimeters to be irradiated. After exposure to the solar simulator, plasmid DNA samples were first treated with DNA repair enzymes (Fpg and T4-endo V) to quantify the number of specific DNA lesions (Fpg-SS, Fpg sensitive sites; T4-endo V-SS, T4-endo V sensitive sites). Electrophoretic migration in agarose gels distinguished supercoiled DNA (form I – FI; free from UV-induced DNA damage) from open-circular relaxed DNA that contained breaks or nicks caused by enzymatic cleavage after recognition of the specific UV-induced DNA damage (form II – FII). Illustration of these experiments and the quantification of CPDs and oxidised DNA damage after exposure to solar simulator are presented in Figure 1.

There was a notable decrease in the amount of DNA damage (DNA from II – FII) in samples that were protected by each of the SPF 30 sunscreens as compared to the vehicle control. Although Fpg-SS was not as efficiently induced as T4-endo V-SS, it was still possible to observe a reduction in the number of oxidised DNA bases in the irradiated samples, as well as a reduction of CPDs, under experimental conditions. DNA-SPF values, as well as the percentage of DNA photoprotection, and the protection provided against the induction of each type of DNA lesion are presented in Table 1.

**Table 1:** DNA photoprotection properties provided by five brands of SPF 30 sunscreens.

Products	DNA-SPF	% DNA photoprotection (95% CI)	% CPD photoprotection (95% CI)	% Oxidised DNA bases photoprotection (95% CI)
**SPF 30 brand 1**	20.0	95.2 (94.6 to 95.7)	95.7 (95.1 to 96.3)	91.1 (90.5 to 91.7)
**SPF 30 brand 2**	20.3	95.1 (94.7 to 95.3)	97.4 (97.2 to 97.6)	76.6 (75.0 to 78.1)
**SPF 30 brand 3**	16.8	94.0 (93.6 to 94.4)	96.0 (95.9 to 96.1)	78.9 (76.0 to 81.8)
**SPF 30 brand 4**	16.9	94.1 (92.9 to 95.2)	96.0 (95.9 to 96.2)	78.6 (70.3 to 86.9)
**SPF 30 brand 5**	20.9	95.2 (94.2 to 96.2)	96.2 (95.4 to 97.0)	87.5 (86.1 to 89.0)

**Legend:** DNA-SPF – Sun Protection Factor for DNA; %DNA photoprotection – percentage of DNA photoprotection; % CPD photoprotection – percentage of protection against CPDs; % oxidised DNA base photoprotection – percentage of protection against oxidised DNA bases; and 95% CI –95% confidence interval of three independent experiments performed in triplicate.

Although the DNA-SPFs were slightly lower than labelled SPFs, all of the tested products appear to offer satisfactory protection against the genotoxic impact induced by simulated exposure to the midday sun at the São Paulo latitude. No statistically significant differences were observed among DNA photoprotection properties provided by the commercial SPF 30 sunscreens that were evaluated in this study (p<0.05).

### DNA Photoprotection Properties Provided by 17 Different Sunscreen Formulations

**Figure 2:**
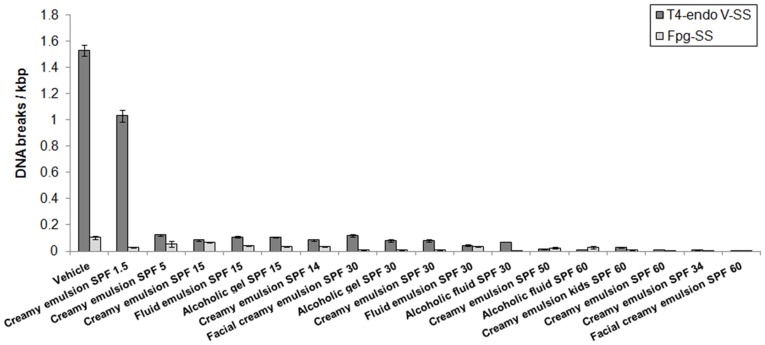
DNA lesions induced by simulated sunlight in the absence (vehicle) or presence of 17 sunscreens. T4-endo V-SS (T4-endo V sensitive sites – CPDs); Fpg-SS (Fpg sensitive sites – oxidised DNA bases). The average and standard deviation in three independent experiments are shown. The experiments were performed in triplicate.

To demonstrate the applicability of the biological dosimeter to the rapid assessment of DNA photoprotection properties of products containing UV filters, the study was extended to evaluate 17 different sunscreen formulations that included creamy emulsion, fluid emulsion, alcoholic gel and alcoholic fluid, as well as 8 SPFs (1.5, 5, 14, 15, 30, 34, 50, and 60). The subsequent quantification of CPDs and oxidised DNA bases that were induced under simulated sunlight conditions are presented in Figure 2.

In general, products with high SPF values offered better protection against the generation of T4-endo V-SS and Fpg-SS. The protection provided by each sunscreen against the induction of these two types of DNA lesions was assessed by quantifying DNA damage, DNA-SPF values, and the percentage of DNA photoprotection. These results are presented in Table 2.

**Table 2:** DNA photoprotection properties provided by 17 different sunscreen formulations.

Products	DNA-SPF	% DNA photoprotection (95% CI)	% CPD photoprotection (95% CI)	% Oxidised DNA bases photoprotection (95% CI)
**Creamy emulsion SPF 1.5**	1.5	35.0 (33.6 to 36.4)	38.4 (35.6 to 41.2)	70.7 (68.4 to 73.0)
**Creamy emulsion SPF 5**	9.2	89.0 (87.8 to 90.2)	92.8 (91.4 to 94.2)	47.8 (33.5 to 61.3)
**Creamy emulsion SPF 15**	9.9	89.9 (89.5 to 90.3)	93.8 (93.4 to 94.2)	54.8 (52.8 to 56.8)
**Fluid emulsion SPF 15**	10.7	90.6 (90.3 to 90.9)	93.4 (92.8 to 94.0)	57.3 (54.1 to 60.5)
**Alcoholic gel SPF 15**	11.4	91.2 (90.9 to 91.5)	93.6 (93.1 to 94.1)	64.1 (62.3 to 65.9)
**Creamy emulsion SPF 14**	13.6	92.6 (92.5 to 92.7)	95.0 (94.7 to 95.3)	64.8 (64.2 to 67.4)
**Facial creamy emulsion SPF 30**	12.5	91.9 (91.1 to 92.7)	92.9 (92.4 to 93.4)	87.9 (85.6 to 90.2)
**Alcoholic gel SPF 30**	17.5	94.2 (93.6 to 94.8)	95.1 (94.7 to 95.5)	87.9 (86.3 to 89.5)
**Creamy emulsion SPF 30**	18.7	94.4 (95.0 to 93.8)	95.3 (94.9 to 95.7)	88.0 (85.9 to 90.1)
**Fluid emulsion SPF 30**	19.1	94.8 (94.6 to 95.0)	96.8 (96.5 to 97.1)	75.9 (74.6 to 77.2)
**Alcoholic fluid SPF 30**	21.8	95.4 (95.2 to 95.6)	95.8 (95.5 to 96.1)	95.0 (94.8 to 95.2)
**Creamy emulsion SPF 50**	40.2	97.5 (97.2 to 97.8)	98.9 (98.8 to 99.0)	76.0 (75.0 to 77.1)
**Alcoholic fluid SPF 60**	41.8	97.5 (96.9 to 98.1)	99.2 (99.0 to 99.4)	72.3 (68.3 to 76.4)
**Creamy emulsion kids SPF 60**	45.7	97.8 (97.7 to 97.9)	98.1 (98.0 to 98.2)	93.3 (92.8 to 93.8)
**Creamy emulsion SPF 60**	100.7	99.0 (99.2 to 98.8)	99.2 (99.1 to 99.3)	96.6 (95.7 to 97.6)
**Creamy emulsion SPF 34**	134.5	99.2 (99.0 to 99.4)	99.5 (99.4 to 99.6)	95.9 (94.9 to 96.9)
**Facial creamy emulsion SPF 60**	160.1	99.4 (99.2 to 99.5)	99.6 (99.5 to 99.7)	96.0 (94.9 to 97.0)

**Legend:** DNA-SPF – Sun Protection Factor for DNA; % DNA photoprotection – percentage of DNA photoprotection; % CPD photoprotection – percentage of protection against CPDs; % oxidised DNA base photoprotection – percentage of protection against oxidised DNA bases; and 95% CI –95% confidence interval of three independent experiments performed in triplicate.

Most of the tested formulations proved to be highly efficient in protecting DNA samples, consistent with their high DNA-SPF values. Some of these values were similar to or even higher than their respective labelled SPFs. Curiously enough, photoprotection against oxidised DNA bases was the most heterogeneous sunscreen attribute. Moreover, high levels of DNA photoprotection could be observed for most products, consistent with the high levels of CPD photoprotection (the most frequent type of sunlight-induced DNA lesion) provided by those sunscreens.

**Figure 3:**
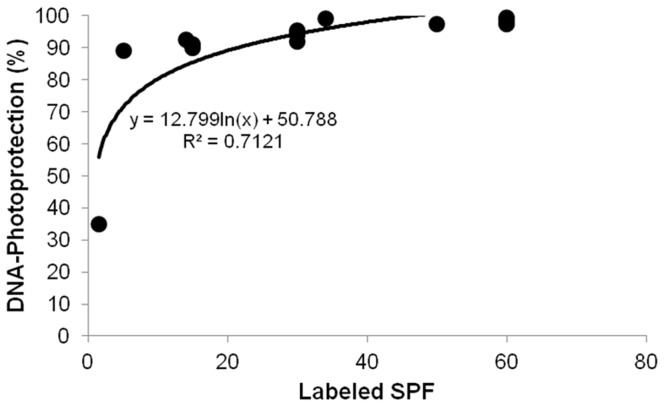
Correlation between the percentages of DNA photoprotection and the labelled SPF values of 17 sunscreen formulations.

To better distinguish the degree of genotoxic protection provided by each sample, the efficacy of the 17 formulations was compared by statistical analysis with subsequent grouping based on their individual efficacies in hindering the generation of DNA damage (for both CPD and oxidised DNA bases). The results of these analyses are presented in Table 3. Figure 3 illustrates the correlation between the percentage of DNA photoprotection (for both CPDs and oxidised DNA bases) and labelled SPF.

**Table 3:** ANOVA and Tukey tests for efficacy discrimination of 17 different sunscreen formulations.

Products	Estimated Average of DNA lesions (CPDs + oxidised DNA bases)	Groups									
**Vehicle**	1.633	A									
**Creamy emulsion SPF 1.5**	1.061		B								
**Creamy emulsion SPF 5**	0.179			C							
**Creamy emulsion SPF 15**	0.155			C	D						
**Fluid emulsion SPF 15**	0.153			C	D						
**Alcoholic gel SPF 15**	0.143			C	D						
**Facial creamy emulsion SPF 30**	0.132			C	D	E					
**Creamy emulsion SPF 14**	0.120				D	E	F				
**Alcoholic gel SPF 30**	0.094					E	F	G			
**Creamy emulsion SPF 30**	0.091					E	F	G			
**Fluid emulsion SPF 30**	0.081						F	G	H		
**Alcoholic fluid SPF 30**	0.075							G	H	I	
**Creamy emulsion SPF 50**	0.041								H	I	J
**Alcoholic fluid SPF 60**	0.040								H	I	J
**Creamy emulsion kids SPF 60**	0.036									I	J
**Creamy emulsion SPF 60**	0.016										J
**Creamy emulsion SPF 34**	0.012										J
**Facial creamy emulsion SPF 60**	0.010										J

**Legend:** Statistical analysis were performed according to the individual efficacy in hindering the generation of DNA damage (CPD and oxidised DNA bases) with subsequent grouping (p<0.05). Increases in the protection efficiency are listed in alphabetical order (A < B < C < D < E < F < G < H < I < J). Statistically significant differences were observed among samples from the different groups, however this was not the case among samples within the same group. Three independent experiments were performed in triplicate.

The results confirm the relationship between DNA photoprotection and labelled SPF and imply that the increased genotoxic protection of a specific product should coincide with an increase in its labelled SPF value, independently of the formulation structure.

## Discussion

Because UV exposure is considered to be the main cause of clinical alterations in sun-exposed skin, an accurate estimate of the photoprotection provided by sunscreen is a major concern in the prevention of the hazardous consequences of prolonged sun exposure. Currently, two major approaches are used in the cosmetic industry when defining the protection properties of sunscreens. These approaches include SPF, which measures erythema induction in the UVB range, and PPD, which measures the oxidation of melanin precursors in the UVA. However, in both cases the measurements consist of skin responses that are not related to the induction of skin cancer [1].

There is an urgent need for complementary methodologies to overcome the existing limitations of the current approaches for measuring photoprotection. Therefore, we propose the use of a highly UV transparent DNA dosimeter to evaluate sunscreen photoprotection against the induction of CPDs and oxidised DNA bases by simulated sunlight. The rationale for choosing both T4-endo V and Fpg enzymes is to facilitate the quantification and qualification of the broad scope of biological protection and the inhibition of different types of natural sunlight-induced DNA damage bestowed by a specific sunscreen.

Another common recurring issue regarding accurate consumer information is the importance of evaluating the photoprotection efficiency of sunscreens in the context of midday sun exposure as a result of prolonged exposure during either occupational or recreational circumstances [1], [24]. The total UV dose and the time of irradiation in the solar simulator that were used in this study were very similar to observed data of natural solar UV radiation around midday on a clear summer day at the São Paulo latitude.

Following the verification of these important parameters, five commercial brands of SPF 30 sunscreen were first irradiated under these conditions, followed by treatment with the DNA repair enzymes (Figure 1). Briefly, all of the tested products efficiently protected against T4-endo V-SS and Fpg-SS induction compared with the unprotected irradiated DNA samples (vehicle), thus demonstrating high levels of DNA photoprotection against simulated solar UV radiation (Table 1). Moreover, all of the samples displayed similar levels of DNA photoprotection with no statistically significant differences (p<0.05).

The study was further extended to evaluate the DNA photoprotection properties of 17 products containing UV filters. It was dramatically clear that, after exposure to the solar simulator, the majority of sunscreens efficiently reduced the amount of T4-endo V-SS and Fpg-SS (Figure 2). High DNA-SPF values were determined, with some values similar to or higher than their corresponding SPF labels (Table 2). Lower DNA-SPFs were observed for products that only listed UVA filters in their formulation, such as Creamy emulsion SPF 1.5 and Creamy emulsion SPF 5. In contrast, Creamy emulsion SPF 14 exhibited a higher DNA-SPF than other SPF 15 sunscreens, including Facial creamy emulsion SPF 30. Interestingly, Creamy emulsion SPF 34 displayed higher protection for both CPDs and oxidised DNA bases when compared to sunscreens with an SPF of 30 and 50 and even higher than some sunscreens with an SPF of 60. Both of these products only have UVB filters in their formulations. The results are in agreement with a previous study in which it was shown that the UVB filter in the formulation was more efficient in protecting against the generation of both CPD and 8-hydroxy-2′-deoxyguanosine than the UVB + UVA combination sunscreens [18]. Together, we have shown that the levels of oxidised DNA base photoprotection provided by the sunscreens examined in this study were lower than the levels of CPDs photoprotection. These results suggest that, in general, sunscreens are more efficient in protecting against the direct formation of pyrimidine dimers than against the generation of oxidised DNA bases, which are formed by indirect mechanisms following the absorption of long UVA wavelengths [25].

With the goal of distinguishing the protective properties of 17 sunscreen formulations by group, a statistical comparison of the individual sunscreen efficacy in reducing the amount of DNA lesions (CPDs and oxidised DNA bases) relative to that observed in unprotected control samples (vehicle) (Table 3) revealed a direct relationship between the labelled SPF value and genotoxic protection, indicating that an increase in DNA photoprotection conferred by a specific product would be in accordance with a higher specified SPF, independent of formulation structure (Figure 3). Furthermore, these results indicate the usefulness of the biosensor for validating the DNA photoprotection properties of various sunscreen formulations.

The main limitation observed in this *in vitro* system resides in the inability to simulate the three-dimensional structural conditions of the skin, as other models using either artificial reconstructed skin [20], [26] or skin explants [1] do. However, these systems also include certain limitations, mainly with regards to the availability of donor tissues and the elevated costs of working with numerous samples or expensive equipment. These methods also require well-trained personnel that are equipped to run specific machinery or the production of large amounts of artificially reconstructed skin with accuracy and reproducibility.

**Figure 4:**
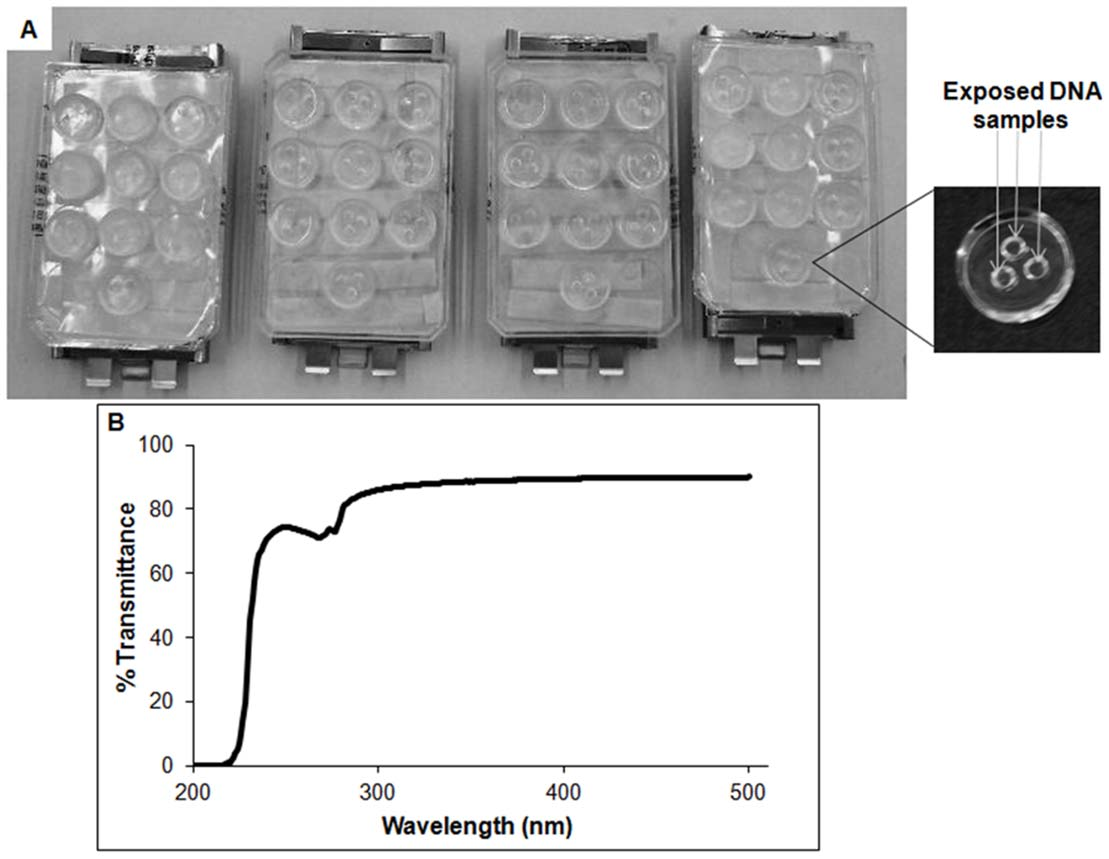
Assessment of the genotoxic protection properties of sunscreens by DNA dosimetry. Exposure of DNA dosimeters to a solar simulator for simultaneously evaluation of DNA photoprotection efficacy of several products containing sunscreen (**A**). The DNA dosimeter transmittance spectrum (**B**).

Furthermore, this system presents certain advantages relevant to applications in the cosmetics industry and is complementary to the current SPF, PPD, and UVA-PF approaches. These advantages include the use of a highly UV-transparent apparatus with a well-defined transmittance spectrum to undertake the exposure of DNA samples to UV sources (Figure 4), the ability to evaluate several products simultaneously in a very simple, easy-to-handle, and cost effective manner, the possibility of testing sunscreens under natural conditions through direct exposure to natural sunlight at different latitudes [27], and the use of various DNA lesion biomarkers (specific enzymes or antibodies) that can be easily standardized. It is important to add that a similar *in vitro* system, employing cultured human cells, for the measurement of DNA damage and cell sensitivity is being developed. DNA repair proficient and deficient cells are being employed which improves the sensitivity of this new system. This work is in progress, and basically validates the usefulness of this approach to assess sunscreen genotoxic photoprotection, as well as it may provide important specific information for people with hypersensitivity to sunlight. Therefore, this work demonstrates that the DNA dosimeter system efficiently measures the amount and nature of specific DNA lesions that are induced by simulated sunlight, as well as the molecular level of photoprotection provided by various commercial products and sunscreen formulations.

## Material and Methods

### Plasmid

Plasmid DNA samples (pCMUT vector, 1762 bp) were purified using the Qiagen Plasmid Maxi Kit (Valencia, CA) with freshly transformed *E. coli* strain *DH10b* [9]. The resulting samples were stored in a TE buffer (10 mM Tris-HCl [pH 8.0], 1 mM EDTA) at −20°C prior to the initiation of experiments.

### The DNA Dosimeter System

The DNA dosimeter apparatus was produced using a special frame developed with the very high UV-transparent Elastomer Syslgard 184 Kit (Dow Corning Corporation, Midland, MI) because this product includes important and adequate features for the exposure of DNA samples to natural or artificial sources of UV radiation [9]. DNA samples were applied in triplicate inside the prototype for the desired exposure times. The DNA dosimeter provides a reliable and reproducible way to calculate the biologically effective dose (BED) of UV radiation by quantifying the amount of specific DNA lesions generated by a specific exposure. This biosensor was previously demonstrated to represent a valid biological dosimeter for assessing photo-induced DNA damage [9], [14], [27]–[28]. Figure 4 illustrates the transmittance spectrum of the DNA dosimeter, as well as its usefulness for collectively evaluating the DNA photoprotection properties of several products that include sunscreen.

### Solar Simulator Irradiation of the DNA Dosimeter in the Presence or Absence of Sunscreen

The DNA dosimeter was exposed to 300,000 J/m^2^ of solar simulated UV radiation using an Atlas Ci400 Xenon Weather-Ometer® solar simulator (Chicago, IL) in the presence or absence of sunscreen. The maximum temperature and relative air humidity measured inside the solar simulator were 40°C and 65%, respectively. The daily UV dose that was used as a reference in the current study was measured under midday sun on a clear-sky summer day in São Paulo, Brazil (23°32′S; 46°38′W) with specific UVB/UVA radiometers (UVB and UVA Radiometers, EKO Instruments Trading Co., Ltda., Japan). Before each irradiation, sunscreens were homogeneously spread onto the surface of the biosensor with a fine brush at a density of 2 mg/cm^−2^, following the recommendations from the COLIPA/CTFA-SA/JCIA/CTFA International Sun Protection Factor (SPF) Test Method Guideline [29]. A weighing method was used to ensure reproducibility, as indicated by this guideline.

### DNA Photoproduct Quantification

After separation by 0.8% agarose gel electrophoresis, the relative amounts of supercoiled and open-circular relaxed plasmid DNA were measured by densitometry analysis (ImageQuant - GE Healthcare, Little Chalfont, UK). Samples with 200 ng of DNA were pre-incubated with 0.8 U of Fpg protein (New England Biolabs, Ipswich, MA) and 70 ng of T4-endo V (produced in this laboratory) prior to discriminating between the different DNA lesions. The samples were then incubated at 37°C for 60 minutes. The enzymes, which were previously assayed at concentrations reaching saturation, were used at concentrations at which no non-specific cleavage is observed. The number of enzyme-sensitive sites per kbp of plasmid DNA was calculated, assuming Poisson distribution that was adapted for this technique, by the following equation:
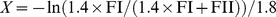
where FI represents the intensity of fluorescence measured in the supercoiled DNA bands, FII represents the intensity in the open-circular relaxed DNA bands, 1.4 is a correction factor to account for the increased fluorescence of ethidium bromide when this compound is bound to relaxed DNA compared to supercoiled DNA, and 1.8 is pCMUT vector size in Kbp [30]. The number of DNA lesions calculated for the irradiated DNA samples were subtracted from the number of breaks observed in the non-irradiated control samples.

### The Sun Protection Factor for DNA (DNA-SPF) and its Percentage of DNA Photoprotection

The calculation of DNA-SPF, adapted from the COLIPA/CTFA-SA/JCIA/CTFA International Sun Protection Factor (SPF) Test Method Guideline [29], was determined as the arithmetical mean of the individual DNA-SPF (DNA-SPF_i_) values obtained from the total number (n) of UV irradiations by the following equation:
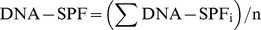
where DNA-SPF_i_ is calculated by the ratio between the total amount of DNA lesions (CPDs + oxidised DNA bases) induced by UV light in each plasmid DNA sample without sunscreen and the total amount of DNA damage verified in each irradiated sample in the presence of sunscreen.

With regards to the variability of the induction of different types of DNA lesions by sunlight, the calculation of the percentage of DNA photoprotection provides a simple and clear approach with which to qualify the biological protection of a specific DNA-SPF. The individual percentage of DNA photoprotection is determined as the weighted arithmetic average of the percentages of protection for both CPDs (CPD photoprotection) and oxidised DNA bases (oxidised DNA bases photoprotection) in each irradiated DNA sample. The total percentage of DNA photoprotection is then determined as the arithmetical mean of individual percentages of DNA photoprotection obtained from the total number of UV irradiations.

### Statistical Analysis

All of the products examined in this study were mutually discriminated by ANOVA and Tukey tests (p<0.05) according to their individual DNA photoprotection efficacy. For the 17 products presenting different formulations and labelled SPF values, these were further separated into groups based on their individual efficacies in hindering the generation of DNA damage (for both CPD and oxidized DNA bases). Increases in the protection efficiency were listed in alphabetical order (A < B < C < D < E < F < G < H < I < J). Statistically significant differences were observed among samples from the different groups, however this was not the case among samples within the same group. Additionally, a correlation analysis was performed between the percentages of DNA photoprotection (for both CPD and oxidised DNA bases) and labelled SPF values of all the 17 products presenting different formulations of sunscreen. Addinsoft XLSTAT (Belmont, MA) was used for the statistical tests.
